# Synthesis of Au-Pd Bimetallic Nanoflowers for Catalytic Reduction of 4-Nitrophenol

**DOI:** 10.3390/nano7090239

**Published:** 2017-08-26

**Authors:** Tao Ma, Feng Liang, Rongsheng Chen, Simin Liu, Haijun Zhang

**Affiliations:** The State Key Laboratory of Refractories and Metallurgy, School of Chemistry & Chemical Engineering, Wuhan University of Science and Technology, Wuhan 430081, China; mataowust@163.com (T.M.); chenrs@wust.edu.cn (R.C.); liusimin@wust.edu.cn (S.L.); zhanghaijun@wust.edu.cn (H.Z.)

**Keywords:** bimetallic nanoparticles, Au-Pd alloy, reduction, 4-nitrophenol

## Abstract

Due to the great potential to improve catalytic performance, gold (Au) and palladium (Pd) bimetallic catalysts have prompted structure-controlled synthesis of Au-Pd nanoalloys bounded by high-index facets. In this work, we prepared Au-Pd bimetallic nanoflowers (NFs) with a uniform size, well-defined dendritic morphology, and homogeneous alloy structure in an aqueous solution by seed-mediated synthesis. The prepared bimetallic NFs were fully characterized using a combination of transmission electron microscopy, Ultraviolet-Visible (UV-vis) spectroscopy, inductively coupled plasma optical emission spectroscopy, and cyclic voltammetry measurements. The catalytic activities of the prepared Au-Pd nanoparticles for 4-nitrophenol reduction were also investigated, and the activities are in the order of Au@Pd NFs > Au-Pd NFs (Au_1_Pd_1_ core) > Au-Pd NFs (Au core), which could be related to the content and exposed different reactive surfaces of Pd in alloys. This result clearly demonstrates that the superior activities of Au-Pd alloy nanodendrites could be attributed to the synergy between Au and Pd in catalysts.

## 1. Introduction

Since Haruta’s seminal work [1,2], well-defined gold nanoparticles (AuNPs) have been developed to achieve chemical transformations, and can be applied in the manufacture of pharmaceuticals, fine chemicals, petroleum processing, and food additives under milder reaction conditions [3,4]. Recently, bimetallic nanoparticles, composed of two different metal elements, have received a great deal of attention, particularly in the field of catalysis, as they have often shown enhanced catalytic performance compared with their corresponding monometallic counterparts, due to alloy effects or synergistic effects [5,6,7,8,9]. Among the various alloy catalysts reported, gold (Au) and palladium (Pd) bimetallic nanoparticles are of particular interest because of their superior activity in low-temperature carbon monoxide oxidation, synthesis of vinyl acetate, direct H_2_O_2_ synthesis, hydrodechlorination of Cl-containing pollutants, hydrodesulfurization of S-containing organics, hydrogenation of hydrocarbons, acetylene trimerization, ethanol oxidation and many other reactions [10,11,12].

Recently, attention has also been focused on anisotropic branched metal nanoparticles that generally exhibit enhanced catalytic activity, which is attributed to their high surface to volume ratio and high-energy active center atoms located at the sharp tips with unsatisfied valency [13,14,15,16]. In our recent work, we synthesized multibranched gold nanostars by a seeded growth method. It was found that the gold nanostars presented better catalytic activity than gold nanocages and gold nanoantennas for the reduction of 4-nitrophenol (4-NP) to its amino derivative 4-aminophenol (4-AP) via sodium borohydride (NaBH_4_) at room temperature [17]. As the degradation of 4-NP is an important reaction in fine chemicals and pharmaceutical industries [18] and can be conveniently tracked by UV-vis absorption spectroscopy, this reaction has been used to study the catalytic efficiency of numerous monometallic and alloy nanocatalysts of different sizes, shapes, and compositions [19].

In this work, we demonstrate that the seed-mediated synthesis of morphology-controlled Au-Pd bimetallic nanoparticles can be conveniently achieved. All resultant Au-Pd nanoflowers exhibited superior catalytic activity for the reduction of 4-nitrophenol as compared to monometallic Au nanoparticles in our lab, and the catalytic activity of the nanoparticles depended on the alloy composition.

## 2. Results and Discussion

### 2.1. Synthesis and Characterization of Au-Pd Nanoflowers

**Scheme 1:**
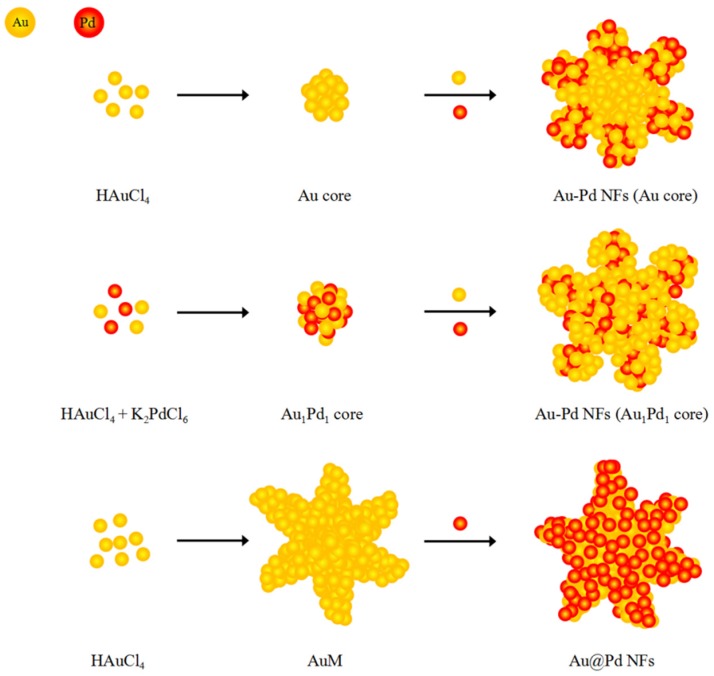
Schematic illustration of the formation of three morphology-controlled Au-Pd nanoflowers (NFs) in this work by seed-mediated growth.

Although there are many routes toward controlling the size, shape, composition and architecture of bimetallic dendritic nanocrystals (e.g., highly branched nanocrystals, stars, and flowers, among others), including seed-mediated growth, galvanic replacement, anisotropic etching of nanocrystals, and aggregation or oriented attachment [8], seed-mediated reduction is the most straightforward method for generating bimetallic nanocrystals [8,11]. Typically, seed-mediated growth requires two processes: (i) the reduction of a metal precursor to form seeds with uniform and relatively small sizes; and (ii) the reduction of another metal on the as-prepared seeds. Therefore, we firstly prepared gold nanospheres (Au core, ~16 nm) [17], gold and palladium bimetallic nanoparticles (Au_1_Pd_1_ core, ~20 nm) [20] and gold nanostars (~40 nm) [17,21] as seeds (Figure S1), then palladium was reduced and deposited on these seed nanocrystals, leading to the formation of three morphology-controlled Au-Pd nanoflowers (Scheme 1).

**Figure 1:**
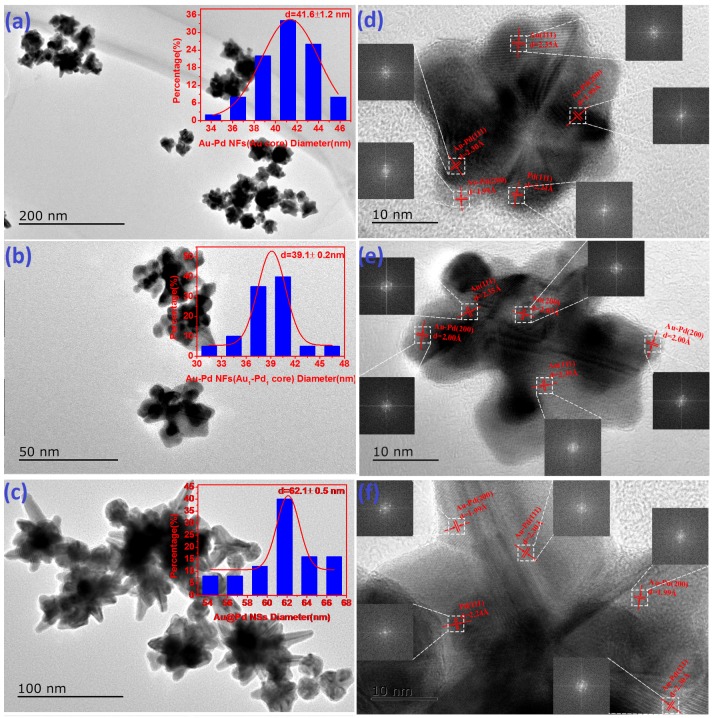
Typical transmission electron microscopy (TEM) images of synthesized nanoflowers in this work: (**a**) Au-Pd NFs (Au core); (**b**) Au-Pd NFs (Au_1_Pd_1_ core); (**c**) Au@Pd NFs; and high-resolution TEM image of (**d**) Au-Pd NFs (Au core); (**e**) Au-Pd NFs (Au_1_Pd_1_ core); (**f**) Au@Pd NFs, the insets indicate the corresponding fast Fourier transform (FFT) patterns obtained in the framed part (dashed line) in the panel. The microstructures were characterized by JEM-2010 UHR microscope (JEOL, Tokyo, Japan).

Two Au-Pd bimetallic nanoflowers (NFs) from gold nanosphere seed (Au core) and gold-palladium bimetallic seed (Au_1_Pd_1_ core) were synthesized by coreduction of HAuCl_4_/K_2_PdCl_6_ mixtures with ascorbic acid in the presence of silver nitrate, the larger Au-Pd bimetallic nanoflowers (Au@Pd NFs) from gold nanostar were synthesized by reduction of K_2_PdCl_6_ with ascorbic acid. Au-Pd NFs (Au core), Au-Pd NFs (Au_1_Pd_1_ core) and Au@Pd NFs all have dendritic shapes with a number of branches, and the average sizes obtained by measuring the length from one branch edge to another one on the opposite side are around 42, 39 and 62 nm respectively (Figure 1a–c). 

In order to verify the formation of the alloyed structure in the prepared NFs, the lattice fringes were further analyzed based on high-resolution transmission electron microscopy (HRTEM) images. The HRTEM images (Figure 1d) and corresponding fast Fourier transform (FFT) patterns (insets in Figure 1d) indicate that Au-Pd NFs (Au core) are obvious crystalline structures with well-defined lattice planes. Interplanar spacings corresponding to the {111} planes of pure metallic Au (JCPDS file number 04-0784) and pure metallic Pd (JCPDS file number 87-0638) were determined, and found to be 0.235 and 0.224 nm, respectively. The measured interplanar distances of Au-Pd NFs (0.230 and 0.199 nm ) show a lower value compared with the interplanar spacing of Au metallic ones, and a higher value compared with the interplanar spacing of Pd metallic ones: 0.235 nm (lattice spacing of Au {111} plane) *>* 0.230 nm *>* 0.224 nm (lattice spacing of Pd {111} plane) and 0.204 nm (lattice spacing of Au {200} plane) *>* 0.199 nm *>* 0.194 nm (lattice spacing of Pd {200} plane), which is evidence of the incorporation of Pd in the Au lattice [22,23,24]. The HRTEM images (Figure 1e,f) also exhibited the {111} or {200} planes of Au-Pd alloy, indicating the formation of the Au-Pd alloy phase in the Au-Pd NFs (Au_1_Pd_1_ core) and Au@Pd NFs because of the close matching between the lattice spacings of Au and Pd.

**Figure 2:**
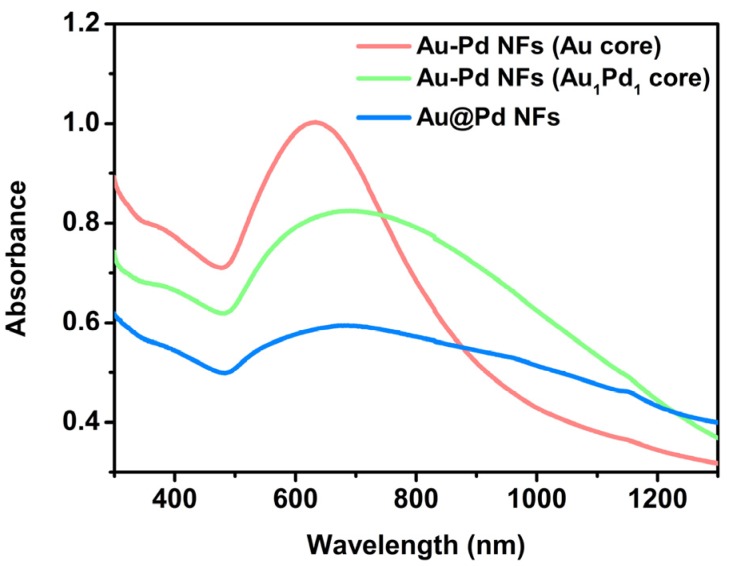
Typical UV-vis spectra of bimetallic nanoflowers solution in aqueous medium at room temperature. The absorption spectra were acquired with a UV-3600 UV-vis-NIR spectrometer (Shimadzu, Kyoto, Japan).

To further confirm the generation of Au-Pd alloy, elemental analyses were performed using inductively coupled plasma optical emission spectroscopy (ICP-OES, IRIS Advantage Duo ER/S spectrometer, Thermo Jarrell Ash, Franklin, MA, USA). Three nanoflower samples were suspended in freshly prepared aqua regia and heated until completely dissolved, and then diluted with double-distilled water [21]. Both Au and Pd elements were able to be detected in the three nanoflower samples, and the mass ratio of Au to Pd is 18:1, 17.6:1, 3.5:1 in Au-Pd NFs (Au core), Au-Pd NFs (Au_1_Pd_1_ core) and Au@Pd NFs, respectively. The synthesized nanoparticles were also characterized by UV-vis spectroscopy as supplement technique, surface plasmon resonance (SPR) peaks of Au-Pd NFs (Au core), Au-Pd NFs (Au_1_Pd_1_ core) and Au@Pd NFs were able to be detected at 633 nm, 685 nm and 695 nm, respectively (Figure 2), which is reasonable for Au-Pd bimetallic nanoparticles [25].

**Figure 3:**
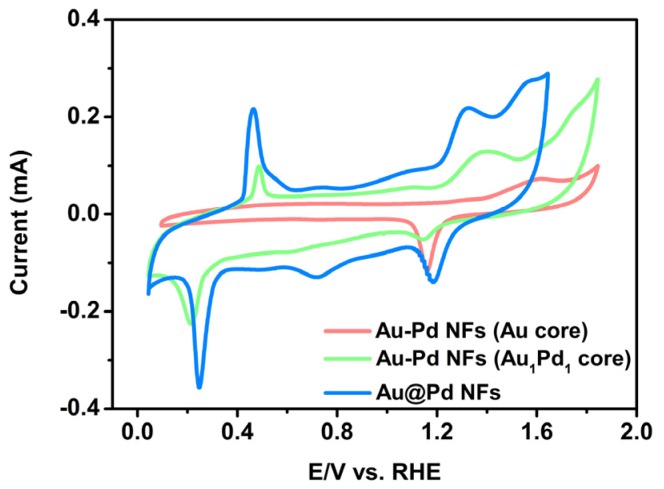
Cyclic Voltammetric (CV) responses (50 mV s^−^^1^) for three nanoflowers in 0.5 M N_2_-saturated H_2_SO_4_ solution up to 1.8 V vs. Reversible Hydrogen Electrode (RHE) and the saturated calomel electrode used as the reference electrode. CV curves were recorded on CHI660e electrochemical workstation (CH Instruments, Shanghai, China).

The cyclic voltammetry (CV) curves were also utilized to evaluate the different nanoparticles. Figure 3 reveals the voltammetric behaviors of Au-Pd nanoflowers in 0.5 M N_2_-saturated H_2_SO_4_ solution [26]. In our case, a combination of the features of both metals was observed, i.e., a more pronounced Au oxide reduction peak around 1.2 V for Au-rich surfaces was observed, while a Pd oxide reduction peak around 0.7 V in the case of Pd-rich surfaces, was also observed [27,28]. The voltammetric features reinforce the earlier conclusions drawn from TEM analyses in Figure 1, and confirm the formation of alloys on the NF surface for the whole stoichiometry range of Au-Pd nanoparticles. From 0.1 to 0.6 V, a pair of well-defined anodic (cathodic) peaks attributed to hydrogen desorption (adsorption), which corresponds to the Pd surface on the alloy because of its unique function in absorption of hydrogen [29].

Moreover, it is curious why Au-Pd nanoflowers were produced in our seed-mediated synthesis. Typically, morphological change could be originally attributed to change in growth kinetics [8,11]. The formation of Au-Pd nanoalloy is based on the coreduction of a stepwise procedure of Pd (IV) to Pd(II) and Pd(II) to Pd(0), and Au(III) to Au(0). Although the PdCl_6_^2^^−^/PdCl_4_^2^^−^ reduction potential (1.288 V) is higher than that of AuCl_4_^−^/Au (1.002 V) and PdCl_4_^2^^−^/Pd (0.591 V), and thus possesses the priority of ascorbic acid reduction, the AuCl_4_^−^/Au and PdCl_4_^2^^−^/Pd conversions directly contribute to the formation of the Au-Pd nanoalloy. The relative high potential of AuCl_4_^−^/Au facilitates the deposition of Au atoms on the core in preference to Pd atoms. After AuCl_4_^−^ was consumed in the solution, the growth of Pd would arise. The further fusion and aggregation of Pd atoms with cores produces the flowerlike structure [8,11,28]. The higher reactivity of Au ions is proved by the inductively coupled plasma (ICP) measurement, which indicated that the Au to Pd mass ratio was able to reach 18:1. The fast mobility of Ag atoms relative to Au atoms, which could promote the random nucleation of Au atoms on the seed to produce the dendritic structure [30,31]. Similar to Au-Rh bimetallic nanoparticles [32,33], the surface free energy for Au is 1.63 J/m^2^, whereas the corresponding value for Pd is 2.04 J/m^2^ [34], which should favor the formation of thermodynamically favorable Au-shell Pd-core structures [35]. The enrichment of the surface with Au has been confirmed by ICP and CV measurements. However, understanding the relationships between reaction conditions and final nanostructures is challenging in our limited examples. A comprehensive examination of how various synthetic parameters influence the bimetallic nanostructure is required.

### 2.2. Catalytic Reduction of 4-Nitrophenol

**Figure 4:**
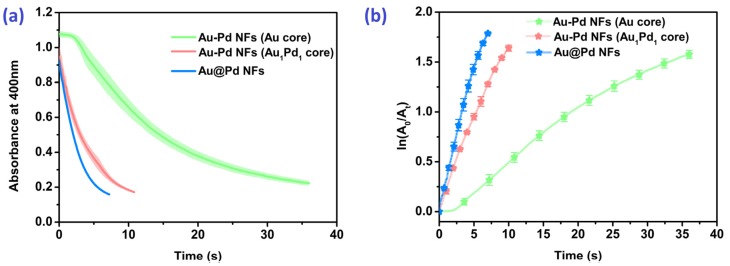
(**a**) The peak intensity at 400 nm for 4-NP as a function of time at room temperature in the presence of NFs. In all cases, the concentrations of 4-NP and NaBH_4_ were 7 × 10^−5^ and 2.1 × 10^−2^ M, respectively, and the concentration of the Au-Pd catalyst was ~8 mg/L; (**b**) the relationships between ln(*A*_0_/*A*_t_) and the reaction time at room temperature in the presence of NFs. The error bars represent standard deviations obtained from three or more trials. These plots were then used to determine the apparent reaction rate constant (*k*_app_). The absorption spectra were acquired with a UV-3600 UV-vis-NIR (ultraviolet-visible-near infrared) spectrometer (Shimadzu, Kyoto, Japan).

Au-Pd NFs were also tested as catalysts for the reduction of 4-nitrophenol with NaBH_4_, according to our reported procedures [17]. After the addition of NaBH_4_, the predominant species 4-nitrophenolate ion is a strong visible absorber with a maximum absorbance at 400 nm. The reduction of 4-NP to 4-AP could be evidenced by a decrease of absorbance to 400 nm [19]. To compare the catalytic activities of NFs, we calculated the reaction rate constants by measuring the intensity (*A*) of the absorption peak at 400 nm with the reaction time and plotting ln(*A*_t_/*A*_0_) versus reaction time t. As shown in Figure 4a, the complete conversion of 4-NP takes less than 1 min. As shown in Figure 4b, a linear relationship between ln(*A*_t_/*A*_0_) and reaction time *t* was obtained for the three NF catalysts in the presence of excess NaBH_4_. The rate constants (*k*_app_), determined by the slopes of the lines, were 15.5, 9.33, and 2.8 min^−1^ for Au@Pd NSs, Au-Pd NFs (Au_1_Pd_1_ core) and Au-Pd NFs (Au core), respectively. This result demonstrates a clear dependence of activity on the chemical composition of NFs. We would also like to stress that our catalyst Au@Pd NS is more active than those reported in the literature [19].

Au-Pd NFs (Au_1_Pd_1_ core, 39 nm) and Au-Pd NFs (Au core, 42 nm) manifest a much higher activity than 40 nm gold nanostars (*k*_app_ = 1.78 min^−1^) and 40 nm gold nanospheres (*k*_app_ = 0.23 min^−1^) [17], showing a strong synergetic effect of the high index facets and Au-Pd alloy [8]. The synergistic effect was further evidenced by increasing the Pd content of bimetallic catalyst, the activity of Au@Pd NSs increased and was able to reach more than 1.7 and 5.5 times as high as that of Au-Pd NFs (Au_1_Pd_1_ core) and Au-Pd NFs (Au core) with almost the same Pd content. The enhanced catalytic activity for 4-NP reduction of Au-Pd NFs (Au_1_Pd_1_ core), compared to Au-Pd NFs (Au core), could be attributed to more exposed reactive surfaces of Pd according to CV curves (Figure 3). Pd by alloying with gold could be favorable to the surface adsorption of the nitrophenol reactant and the reducing agent on the catalyst to follow the Langmuir-Hinshelwood mechanism, and the modification of electronic property of nanoalloys [36,37]. To better understand the synergistic effect in 4-NP reduction by Au-Pd alloy, computational calculation about the binding energy between the reactants adsorbed on the surface of the nanoalloy and the electron transfer effect between Pd and Au atoms is in progress. It is also noteworthy that the reactants could significantly change the morphology and topology of nanocatalysts [38,39], which requires specific attention in future works.

## 3. Materials and Methods 

### 3.1. Synthesis of Au-Pd NFs(Au Core and Au_1_Pd_1_ Core)

Au-Pd NFs were prepared by a seed-mediated growth method. The seed solutions of Au and Au_1_Pd_1_ nanoparticles were prepared according to the reported procedures [17,20], and were then added to 29.7 mL of double distilled water under moderate stirring for 30 s. After that, 90 μL of AgNO_3_ (10 mM), 2 mL of K_2_PtCl_6_ (0.5 mM) and 0.1 mL of chloroauric acid (10 mM) were added sequentially. After sufficient dispersion, 0.5 mL of ascorbic acid (0.1 M) was added to the mixture, the Au-Pd NFs (Au core) and Au-Pd NFs (Au_1_Pd_1_ core) were produced severally.

### 3.2. Synthesis of Au@Pd NFs

In a typical synthesis of Au@Pd NFs, 200 mL of HAuCl_4_ (10 mM) was diluted with 10 mL of water, then 30 μL of AgNO_3_ (10 mM) was added. After the resulting solution was thoroughly mixed, 40 μL of ascorbic acid (100 mM) was quickly added, and the mixture was stirred vigorously for 20 s at room temperature, then the gold multibranched nanoparticles (AuM) were produced as a seed solution. For the subsequent growth of the Pt shell, 200 μL of K_2_PtCl_6_ (10 mM) was added into 10 mL of AuM colloids under stirring, followed by addition of 40 μL of ascorbic acid (100 mM). The color of the mixture changed from blue to dark gray, and Au@Pd NFs was produced. 

## 4. Conclusions

We have developed a reproducible and facile method for the preparation of morphology-controlled Au-Pd bimetallic dendritic nanoflowers using seed mediated reduction. The prepared three NFs were characterized by UV-Vis, TEM, CV and HRTEM. The resultant Au-Pd nanodendrites exhibited a narrow size distribution and well-defined morphology, and the mass ratio of Au to Pd was able to be reduced from 18:1 to 3.5:1, detected by inductively coupled plasma optical emission spectroscopy. Three Au–Pd nanoflowers exhibited excellent catalytic activities toward the reduction of 4-NP, and the catalytic performances could be effectively tuned by varying the ratio of Au to Pd and the micro-structure of the surfaces.

## Supplementary Materials

The following are available online at www.mdpi.com/2079-4991/7/9/239/s1, Figure S1: Typical transmission electron microscopy (TEM) images of synthesized seeds in this work.
