# Supplementary material for: Synthesis of Au-Pd Bimetallic Nanoflowers for Catalytic Reduction of 4-Nitrophenol
Source: Nanomaterials (Basel). 2017 Aug 26;7(9):239. doi: 10.3390/nano7090239 (PMC5618350; doi:10.3390/nano7090239)
Supplement: Supplementary file 1 [file nanomaterials-07-00239-s001.docx]

Supplementary Materials: Synthesis of Au-Pd Bimetallic Nanoflowers for Catalytic Reduction of 4-Nitrophenol

Tao Ma, Feng Liang *, Rongsheng Chen, Simin Liu and Haijun Zhang


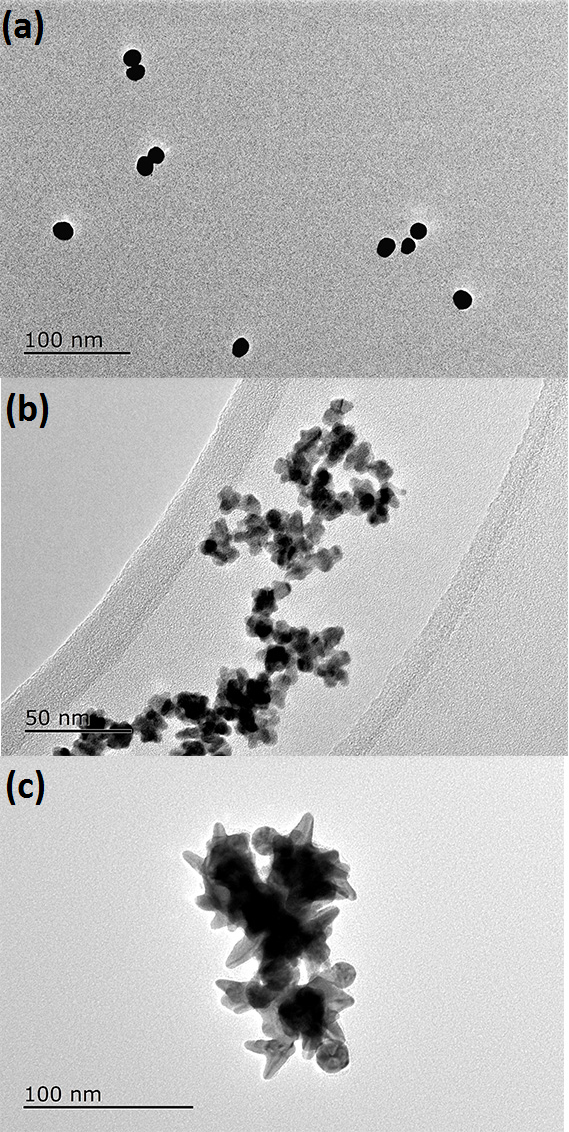


**Figure S1.** Typical transmission electron microscopy (TEM) images of synthesized seeds in this work: (**a**) gold nanosphere (Au core, ~16 nm); (**b**) gold and palladium bimetallic nanoparticles (Au_1_Pd_1_ core, ~20 nm); (**c**) gold nanostar (~40 nm). The microstructures were characterized by TEM (JEM-2010 UHR, JEOL, Tokyo, Japan).
